# Update DVO-Leitlinie 2023 „Prophylaxe, Diagnostik und Therapie der Osteoporose bei postmenopausalen Frauen und bei Männern ab dem 50. Lebensjahr“ – Was ist neu für die Rheumatologie?

**DOI:** 10.1007/s00393-024-01495-x

**Published:** 2024-03-21

**Authors:** Alexander Pfeil, Uwe Lange

**Affiliations:** 1 Klinik für Innere Medizin III, Funktionsbereich Rheumatologie und Osteologie, Universitätsklinikum Jena, Am Klinikum 1, 07747 Jena, Deutschland; 2 Kerckhoff-Klinik GmbH, Abteilung Rheumatologie und Klinische Immunologie, Justus-Liebig-Universität Gießen, Benekestraße 2–8, 61231 Bad Nauheim, Deutschland

**Keywords:** Osteoporose, DVO-Leitlinie 2023, Entzündlich-rheumatische Erkrankungen, Osteospezifische Therapie, Osteoporosis, DVO Guideline 2023, Inflammatory rheumatic diseases, Osteospecific therapy

## Abstract

Im Oktober 2023 veröffentlichte der Dachverband der deutschsprachigen wissenschaftlichen osteologischen Gesellschaften e. V. (DVO) die überarbeitete Leitlinie zur „Prophylaxe, Diagnostik und Therapie der Osteoporose bei postmenopausalen Frauen und bei Männern ab dem 50. Lebensjahr“. Dieser Übersichtsartikel geht auf die Neuerungen der Leitlinie und deren Relevanz in der Betreuung von Betroffenen mit entzündlich-rheumatischen Erkrankungen ein.

Eine zentrale Änderung der Leitlinie stellt die Umstellung des 10-Jahres-Frakturrisikos auf das 3‑Jahres-Frakturrisiko dar. Die Basisdiagnostik wird aktuell ohne definierte Frakturschwelle durchgeführt. Als weitere Schlüsselneuerung sind die Therapieschwellen für die spezifisch osteologische Therapie mit 3 % bis < 5 %, 5 % bis < 10 % und ab 10 % für Wirbelkörper- sowie Schenkelhalsfrakturen zu nennen. Bei einem 3‑Jahres-Frakturrisiko > 10 % ist primär eine osteoanabole Therapie durchzuführen und eine antiresorptive Therapie wird an die osteoanabole Therapie angeschlossen. Weiterführend sollten Patientinnen und Patienten mit einer Osteoporose sowie einer länger andauernden Glukokortikoidtherapie primär osteoanabol mittels Teriparatid behandelt werden. Zusammenfassend reflektieren die Änderungen der DVO-Leitlinie die aktuellen wissenschaftlichen Studienerkenntnisse in der Osteologie und führen zu einer detaillierten Differentialtherapie der Osteoporose.

## Abstract (en)

In October 2023, the organization of the German-speaking scientific osteological societies (DVO) published the revised guideline on the “Prophylaxis, diagnosis and treatment of osteoporosis in postmenopausal women and in men aged over 50.” This review article reflects the new features of the guideline and their relevance in the care of patients with inflammatory rheumatic diseases.

A key innovation is the change from the 10-year fracture risk to the 3‑year fracture risk. Basic diagnostics are currently performed without a defined fracture threshold. Treatment thresholds for specific osteological therapy constitute another key innovation, defined as 3% to < 5%, 5% to < 10%, and from 10% for vertebral body and femoral neck fractures. If the 3‑year fracture risk is > 10%, osteoanabolic therapy should primarily be carried out and antiresorptive therapy is initiated following osteoanabolic therapy. In addition, patients with osteoporosis and prolonged glucocorticoid therapy should primarily be treated osteoanabolically with teriparatide. In summary, the changes to the DVO guideline reflect the latest scientific study findings in osteology and lead to detailed differential therapy for osteoporosis.

Im Oktober 2023 wurde die aktualisierte Leitlinie des Dachverbandes der deutschsprachigen wissenschaftlichen osteologischen Gesellschaften e. V. (DVO) zur „Prophylaxe, Diagnostik und Therapie der Osteoporose bei postmenopausalen Frauen und bei Männern ab dem 50. Lebensjahr“ veröffentlicht [5]. Im Rahmen des folgenden Übersichtsartikels sollen die Neuerungen der DVO-Leitlinie 2023 dargestellt werden, welche in der Betreuung von Patientinnen und Patienten mit entzündlich-rheumatischen Erkrankungen als wichtig anzusehen sind.

## Risikofaktoren für eine Osteoporose

Unverändert zur Leitlinie aus dem Jahr 2017 werden die rheumatoide Arthritis, axiale Spondyloarthritis (Spondylitis ankylosans) und der systemische Lupus erythematodes als Risikofaktoren für eine Osteoporose ausgewiesen. Im Hinblick auf die medikamentösen Therapien wird unverändert die systemische Applikation von Glukokortikoiden unter Berücksichtigung der Dosis und der Anwendungsdauer mit einem signifikanten Anstieg des Frakturrisikos im Bereich der Wirbelsäule und Hüfte dargestellt.

## Knochenmineraldichtemessung und Bestimmung des Frakturrisikos

Als Standardknochenmineraldichtemessverfahren wird weiterhin die Dual-Energy-X-ray-Absorptiometrie(DXA)-Methode favorisiert. Entsprechend den aktuellen Empfehlungen sollte eine DXA-Knochenmineraldichtemessung an der Lendenwirbelsäule und am proximalen Femur beidseits (Schenkelhals und Femur gesamt) durchgeführt werden. In die Beurteilung der Therapieschwelle geht aber nur der T‑Score der Hüfte (gesamt) ein.

Hinsichtlich der quantitativen Computertomographie ergibt sich eine Änderung, dass beim Vorhandensein einer Knochenmineraldichtemessung mittels quantitativer Computertomographie (mindestens zwei auswertbare Wirbelkörper im Bereich des Brustwirbelkörpers 12 bis Lendenwirbelkörper 4) und einer messbaren trabekulären Knochenmineraldichte < 80 mg/ml Hydroxylapatitgehalt diese Daten zur Beurteilung des Frakturrisikos herangezogen werden können.

## Frakturrisiko

Als eine der wichtigsten Neuerungen ist zu nennen, dass die Vorhersage des 10-Jahres-Frakturrisikos verlassen wird und durch das 3‑Jahresrisiko für Schenkelhals- und Wirbelkörperfrakturen ersetzt wird.

## Indikationen zur Basisdiagnostik

Im Gegensatz zur Leitlinie aus dem Jahr 2017 wird in der aktuellen Version keine Frakturrisikoschwelle (DVO-Leitlinie 2017: 20 % osteoporotisches Frakturrisiko bezogen auf 10 Jahre) zur Diagnostik definiert [4, 5]. Beim Vorliegen entsprechender Risikofaktoren (z. B. Wirbelkörper- oder Schenkelhalsfraktur) sollte bei postmenopausalen Frauen und Männern ab dem 50. Lebensjahr eine entsprechende osteologische Basisdiagnostik durchgeführt werden. Aufgrund des geringen Frakturrisikos vor dem Auftreten der Menopause und bei Männern vor dem 50. Lebensjahr wird generell keine osteologische Basisdiagnostik empfohlen [5], wenngleich unter Berücksichtigung der klinischen Gesamtsituation im Sinne eines „Case Findings“ eine osteologische Basisdiagnostik initiiert werden kann [5]. Aus rheumatologischer Sicht sind als besondere Risikofaktoren für die Einleitung einer Diagnostik die Erkrankungsbilder rheumatoide Arthritis, Spondyloarthritis und systemischer Lupus erythematodes sowie die systemische (orale) Applikation von Glukokortikoiden zu nennen.

Bei Frauen im Alter von > 70 Jahren wird bei einer entsprechenden therapeutischen Konsequenz eine Knochenmineraldichtemessung ohne das Vorhandensein von Risikofaktoren durchgeführt.

## Osteologische Basistherapie

Unverändert zur DVO-Leitlinie 2017 wird die Kalziumaufnahme von mindestens 1000 mg je Tag und eine Vitamin-D3-Substitution von täglich 800–1000 IE empfohlen [5].

## Therapieschwellen zur spezifischen osteologischen Therapie

**Figure 1:**
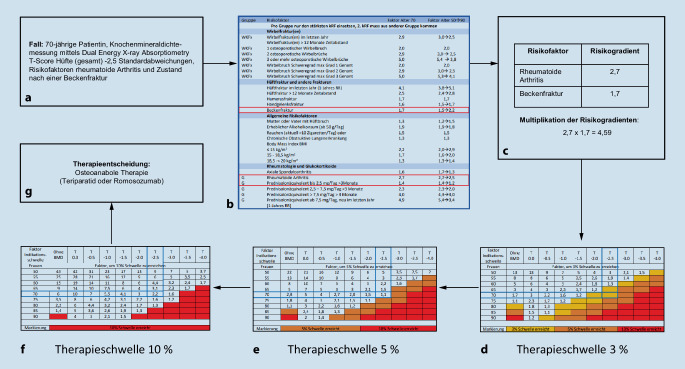

Neu werden in der DVO-Leitlinie 2023 drei Therapieschwellen zur spezifischen osteologischen Therapie bezogen auf das 3‑Jahres-Frakturrisiko für Wirbelkörper- und Schenkelhalsfrakturen genannt: 3 % bis < 5 %, 5 % bis < 10 % und ab 10 %. Die Berechnung der Therapieschwellen erfolgt anhand der in der Leitlinie angegebenen Tabellen (siehe Abb. 1). Perspektivisch soll im Frühjahr 2024 ein digitaler Rechner zur Bestimmung der Therapieschwellen implementiert werden.

Des Weiteren ermöglicht die Leitlinie bei Patientinnen und Patienten mit einem hohen Frakturrisiko im Kontext von starken Frakturrisikofaktoren unabhängig von den oben genannten drei Therapieschwellen eine frühzeitigere Einleitung einer spezifischen osteologischen Therapie. Eine klare Definition dieser Hochrisikosituation wird in der Leitlinie nicht vorgenommen.

## Spezifische osteologische Therapie

Eine Übersicht zur Therapiedauer der spezifischen osteologischen Therapie entsprechend der DVO-Leitlinie 2023 gibt Tab. 1. Bei einem 3‑Jahres-Frakturrisiko von 3 % bis < 5 % wird die Durchführung einer spezifischen osteologischen Therapie empfohlen. Hier sollten primär antiresorptive Medikamente eingesetzt werden. Eine osteoanabole Therapie kann bei einem Frakturrisiko zwischen 5 % bis < 10 % erwogen werden, wobei bei einem Frakturrisiko ab 10 % eine osteoanabole Therapie einzuleiten ist. Die osteoanabole Therapie wird als Sequenztherapie durchgeführt, d. h. an die osteoanabole Therapie schließt sich eine antiresorptive Therapie zum Erhalt der Knochenmineraldichte an. Als osteoanabole Therapien können Teriparatid oder Romosozumab (nach Indikationszulassung) eingesetzt werden. Als Indikation zur Applikation von Teriparatid sind die postmenopausale Osteoporose, die Osteoporose bei Männern als auch die Glukokortikoidtherapie assoziierte Osteoporose jeweils mit einem hohen Frakturrisiko zu nennen. Demgegenüber kann Romosozumab nur bei postmenopausaler Osteoporose mit einem deutlich erhöhten Frakturrisiko eingesetzt werden. Weiterführend konnte in einer Post-hoc-Analyse gezeigt werden, dass in der Therapiesequenz Romosozumab-Denosumab ein Anstieg der Knochenmineraldichte im Bereich der Lendenwirbelsäule nach 2 Jahren von 16,6 % im Vergleich zur Therapiesequenz Romosozumab-Alendronat mit 15,2 % nachgewiesen werden konnte [3].

**Table 1:** 

Therapieprinzip	Wirkstoff	Behandlungsdauer	Zulassungsstatus	Folgetherapie nach Beendigung der initialen Therapie
*Antiresorptive Therapie*	Bisphosphonate	3–5 Jahre	*Alendronat, Risedronat und Zoledronat:* Postmenopausale Frauen und Osteoporose bei Männern *Ibandronat:* Postmenopausale Frauen	–
	Denosumab	Unbefristet	Postmenopausale Frauen und Osteoporose bei Männern	Indiziert (z. B. Zoledronat 5 mg, ein bis zwei Applikationen, 1. Gabe am Ende des Denosumab-Intervalls, 2. Gabe in Abhängigkeit von Knochenumbaumarkern und Knochenmineraldichte, bei fehlenden Knochenumbaumarkern Applikation Zoledronat zu Monat 6 und 12 nach letzter Denosumab-Gabe)
	Raloxifen	Bis zu 8 Jahre	Postmenopausale Frauen	–
*Osteoanabole Therapie*	Romosozumab	1 Jahr (pro Therapiezyklus)	Postmenopausale Frauen	Antiresorptive Therapie
	Teriparatid	2 Jahre (Lebenszeit)	Postmenopausale Frauen und Osteoporose bei Männern mit hohem Frakturrisiko	Antiresorptive Therapie

Entsprechend dem Zulassungsstatus und den Therapieempfehlungen darf eine Teriparatid-Therapie nur einmalig durchgeführt werden, demgegenüber kann die Therapiesequenz Romosozumab-antiresorptive Substanz beliebig wiederholt werden.

An dieser Stelle ist anzumerken, dass Romosozumab nur zur Behandlung der postmenopausalen Osteoporose zugelassen ist. In den Zulassungsstudien von Romoszumab wurden numerisch mehr ischämische kardiovaskuläre Ereignisse (0,8 %), ischämische cerebrovaskuläre Ereignisse (0,8 %) und kardiovaskulär bedingter Tod (0,8 %) im Vergleich zu Alendronat (ischämische kardiovaskuläre Ereignisse: 0,3 %, ischämische cerebrovaskuläre Ereignisse 0,3 % und kardiovaskulär bedingter Tod 0,6 %) nachgewiesen [9], sodass Romosozumab nicht bei Patienten mit einem Myokardinfarkt bzw. einen ischämisch bedingten Schlaganfall eingesetzt werden sollte. In diesem Zusammenhang ist vor dem Therapiebeginn mit Romosozumab das kardiovaskuläre Risiko zu evaluieren und in Kontext zum Frakturrisiko zu setzen.

In Bezug auf die wichtigen Kontraindikationen für Teriparatid sind die chronische Niereninsuffizienz im Stadium Chronic Kidney Disease (CKD) G4 und G5 [8] sowie die Hyperkalzämie, der Hyperparathyreoidismus, der Morbus Paget und ossäre Malignome inklusive Skelettmetastasen zu nennen.

Zusätzlich wurden Risikofaktorengradienten pro Risikofaktor definiert, welche die Therapieschwelle in Hinblick auf eine antiresorptive bzw. osteoanabole Therapie erniedrigen können. Beim Vorliegen mehrerer Risikofaktoren sollten die Risikofaktorengradienten der zwei stärksten Risikofaktoren multipliziert werden, wobei innerhalb der Gruppe „Wirbelkörperfrakturen“, „Rheumatologie und Glukokortikoide“ (Ausnahme axiale Spondyloarthritis) sowie „Sturzrisiko assoziierte Risikofaktoren/Geriatrie“ keine Multiplikation von zwei Risikofaktoren vorgenommen werden sollte, damit das Frakturrisiko nicht überschätzt wird. In Tab. 2 wird eine Übersicht zu den Risikofaktoren gegeben.

**Table 2:** 

	Risikofaktoren
*Wirbelkörperfraktur(en)*	Wirbelkörperfrakturen im letzten Jahr
	Anzahl der Wirbelkörperfrakturen und Schweregrad der Wirbelkörperfrakturen
*Hüftfrakturen und andere Frakturen*	Hüftfraktur
	Humerusfraktur
	Handgelenksfraktur
	Beckenfraktur
*Allgemeine Risikofaktoren*	Alkoholkonsum (> 50 g/Tag)
	Nikotinabusus (> 10 Zigaretten/Tag)
	Chronisch obstruktive Lungenerkrankung
	Untergewicht (Body-Mass-Index < 20 kg/m^2^)
*Rheumatologische Risikofaktoren und Glukokortikoide*	Rheumatoide Arthritis
	Axiale Spondyloarthritis
	Glukokortikoideinnahme
*Sturzrisiko assoziierte bzw. geriatrische Risikofaktoren*	Sturz im letzten Jahr
	Chronische Hyponatriämie
	Depression bzw. Einnahme von Antidepressiva
	Immobilität
	Epilepsie
	Multiple Sklerose
	Morbus Alzheimer/Demenz
	Morbus Parkinson
	Schlaganfall
	Opioide
	Timed-up-and-go-Test > 12 Sek
*Endokrinologische Risikofaktoren*	Diabetes mellitus Typ 1
	Diabetes mellitus Typ 2
	Primär Hyperparathyreoidismus
	Hyperthyreose
*Weitere Erkrankungen mit Risikofaktoren bzw. Medikamente*	Chronische Herzinsuffizienz
	Chronische Niereninsuffizienz im Stadium CKD 3A, 3B und 4
	Monoklonale Gammopathie unklarer Signifikanz
	Einnahme von Protonenpumpeninhibitoren länger als 3 Monate
*Trabekular Bone Score (TBS)*	TBS abgestuft zwischen −1,0 Standardabweichung bis −2,5 Standardabweichung

Als rheumatologische Risikofaktoren mit einem Risikogradienten, welche mit einer Änderung der Therapieschwelle assoziiert sind, werden die axiale Spondyloarthritis, die rheumatoide Arthritis sowie Glukokortikoide gewichtet entsprechend der täglichen Dosis in Prednisolonäquivalent und Therapiedauer aufgeführt. Der systemische Lupus erythematodes stellt einen Risikofaktor für eine Osteoporose dar, dennoch wird der systemische Lupus erythematodes nicht als Risikofaktor mit Risikogradient aufgeführt. Nach Ansicht der Leitlinienautoren weist der systemische Lupus erythematodes eine geringe Prävalenz in der Allgemeinbevölkerung auf und das Erkrankungsbild wird zumeist an rheumatologisch-osteologischen Schwerpunktzentren mit entsprechender Kompetenz betreut. Zusätzlich wird in der Leitlinie dargestellt, dass Patientinnen und Patienten mit einem systemischen Lupus erythematodes häufig ein Alter unter 50 Jahren sowie ein hohes Risiko für eine Osteoporose aufgrund der Entzündungsaktivität und der Einnahme von Glukokortikoiden aufweisen. Aus diesen Gründen sollte als Einzelfallentscheidung vor dem 50. Lebensjahr eine osteologische Basisdiagnostik durchgeführt und gegebenenfalls eine spezifische osteologische Therapie eingeleitet werden.

Neu wird in der Leitlinie die osteoanabole Therapie bei einer Glukokortikoidtherapie definiert. Bei Betroffenen mit einem hohen Frakturrisiko und einer Glukokortikoidtherapie mit Prednisolon (> 5 mg Prednisolonäquivalent/Tag über mehr als 3 Monate) sollte primär eine osteoanabole Therapie mittels Teriparatid durchgeführt werden.

## Pausierung der spezifischen osteologischen Therapie

Als einzige Substanzgruppe wird für die Bisphosphonate die Evaluierung einer Therapiepause bei einem Abfall des Risikos unter die Therapieschwelle in der aktualisierten Leitlinie diskutiert. Bei einem hohen Frakturrisiko sollte die Therapie fortgeführt werden. Eine Therapiepause kann nach einer Therapiedauer mit Alendronat nach 5 Jahren bzw. Zoledronat nach 3 Jahren diskutiert werden, wenn keine osteoporotisch bedingten Frakturen vor und während der Therapie vorlagen. Zusätzlich sollte der T‑Score am Femurhals > −2,5 Standardabweichungen betragen und keine weiteren Risikofaktoren vorhanden sein. Nach der Pausierung der Bisphosphonattherapie wird eine Überwachung der Knochenumbauparameter als auch der Knochenmineraldichte mittels DXA-Methode empfohlen.

In der DVO-Leitlinie 2023 wird noch einmal detailliert darauf hingewiesen, dass Denosumab ohne eine anschließende antiresorptive Therapie nicht beendet werden sollte, da sonst ein signifikanter Anstieg des Frakturrisikos (vertebrale Frakturen vor der Denosumab-Therapie 16,4 %; vertebrale Frakturen im Rahmen der Denosumab-Therapie 2,2 % versus vertebrale Frakturen nach Absetzen der Denosumab-Therapie 10,3 %) zu verzeichnen ist [1]. Aus diesem Grund sollte Denosumab ohne zeitliche Begrenzung fortgeführt werden. Dies spielt vor allem bei Betroffenen mit einer chronischen Niereninsuffizienz eine wichtige Rolle, da alle Bisphosphonate bzw. Raloxifen bei einer chronischen Niereninsuffizienz im Stadium CKD G4 und G5 sowie teilweise im Stadium G3 nicht zugelassen sind [8].

Ist eine Pausierung bzw. ein Absetzen der Denosumab-Therapie notwendig, muss eine intravenöse Anschlusstherapie mit Zoledronat (1 bis 2 Applikationen in Abhängigkeit vom Verlauf der Knochenmineraldichte und der Knochenumbaumarker) durchgeführt werden [5]. Die erste Gabe von Zoledronat erfolgt am Ende des Denosumab-Intervalls [5]. Ist die Gabe von Zoledronat nicht möglich, kann alternativ Alendronat verordnet werden. Nach der Beendigung der Denosumab-Therapie muss eine Kontrolle der Knochenumbaumarker im Monat 3 sowie Monat 6 nach Therapiebeendigung durchgeführt werden und in Abhängigkeit der Befunde ist gegebenenfalls eine frühzeitigere weitere Applikation von Zoledronat indiziert. Bei einer fehlenden Bestimmung der Knochenumbauparameter wird eine Applikation von Zoledronat entsprechend 6 und 12 Monate nach der letzten Denosumab-Applikation vonseiten der Leitlinie empfohlen [5, 10, 11].

## Welche Limitationen sind in Hinblick auf die DVO-Leitlinie 2023 zu nennen?

Aus osteologischer Sicht bzw. rheumatologischer Sicht spielt die Behandlung der Osteoporose bei einer Niereninsuffizienz bzw. die Glukokortikoid-induzierte Osteoporose eine entscheidende Rolle.

Im Hinblick auf eine Einschränkung der Niereninsuffizienz ist anzumerken, dass neben einer renalen Osteopathie auch eine Osteoporose auftreten kann [8]. Hierbei sind die Begriffe renale Osteopathie und Osteoporose nicht gleichzusetzen, da die Osteoporose mit einer Verminderung des Knochenmineralsalzgehaltes und einer Mikrostrukturveränderung des Knochens mit einem erhöhten Frakturrisiko verbunden ist und die renale Osteopathie durch eine Hyperkalzämie, Hypocholesterinämie, Hyperparathyreoidismus, Calcitriolmangel sowie eine erhöhte FDG-23-Konzentration gekennzeichnet ist [2].

Patientinnen und Patienten mit einer zunehmenden Einschränkung der Nierenfunktion weisen ein erhöhtes Frakturrisiko auf [7]. In diesem Zusammenhang ist auch durch entsprechende Leitlinienempfehlungen die Therapie einer Osteoporose bei einer eingeschränkten Nierenfunktion darzustellen.

Äquivalent zu den vorhergehenden Leitlinien bezieht die aktualisierte Version der DVO-Leitlinie keine detaillierte Stellung hinsichtlich der Behandlung einer Glukokortikoid-induzierten Osteoporose und deren Differentialtherapie. Hier wird auf die Empfehlungen der Deutschen Gesellschaft für Rheumatologie zum Management der Glukokortikoid-induzierten Osteoporose aus dem Jahr 2021 verwiesen [6].

Diese beiden aus osteologisch-rheumatologischer Sichtweise wichtigen Themen werden in der aktuellen DVO-Leitlinie 2023 erneut nicht adressiert.

## Zusammenfassung

Zusammenfassend sind als wichtige Erneuerungen der DVO-Leitlinie 2023 die Absenkung des Frakturrisikos auf die 3‑Jahresschwelle und die Durchführung einer Sequenztherapie zur Behandlung der Osteoporose mit einer primären osteoanabolen, gefolgt von einer antiresorptiven Therapie zu nennen.

## Fazit für die Praxis

- Berücksichtigung des T‑Scores an der Hüfte (gesamt, gemessen mittels DXA-Knochenmineraldichtemessung) zur Bestimmung der Therapieschwelle
- Spezifische Therapieschwellen in Abhängigkeit des 3‑jährigen Frakturrisikos
- Primäre osteoanabole Therapie bei einem 3‑Jahres-Frakturrisiko ab 10 %

## Abkürzungen

CKD: Chronic Kidney Disease
DVO: Dachverband Osteologie e. V.
DXA: Dual-Energy-X-ray-Absorptiometrie
